# Design and Analysis of a Smart Watch Antenna Operating in the 2.4 GHz Band

**DOI:** 10.3390/s26123921

**Published:** 2026-06-20

**Authors:** Łukasz Januszkiewicz, Remigiusz Danych, Maciej Łaski, Kornelia Bendzel

**Affiliations:** 1 Institute of Electronics, Faculty of Electrical, Electronic, Computer and Control Engineering, Lodz University of Technology, 90-924 Lodz, Poland; 246953@edu.p.lodz.pl; 2 GlobalRnD sp. z o.o., 91-341 Lodz, Poland; danych@globalrnd.pl (R.D.); laski.maciej@globalrnd.pl (M.Ł.)

**Keywords:** antenna, FDTD, wearable electronics, WBAN

## Abstract

This paper presents the design of an inverted-F antenna intended for integration into a smartwatch operating in the 2.4 GHz band. The antenna design addresses spatial constraints imposed by the device’s miniaturized form factor and the proximity of electronic components, including the printed circuit board, display, and battery. The influence of the user’s body on the antenna’s performance characteristics was considered during the design phase through numerical simulations employing the Finite-Difference Time-Domain (FDTD) method with a heterogeneous human body model. Simulation results and measurements of a fabricated prototype antenna are presented, demonstrating satisfactory performance in terms of impedance matching with VSWR below 1.5 in the whole band and gain of −1 dBi.

## 1. Introduction

With the dynamic development of electronics, there is a continuous need to miniaturize components and to integrate increasingly complex functions into small-sized devices. This trend is reflected in the development of smart wearable devices, which are gaining popularity due to their ability to monitor various user’s parameters and to interact with the environment.

**Figure 1:**
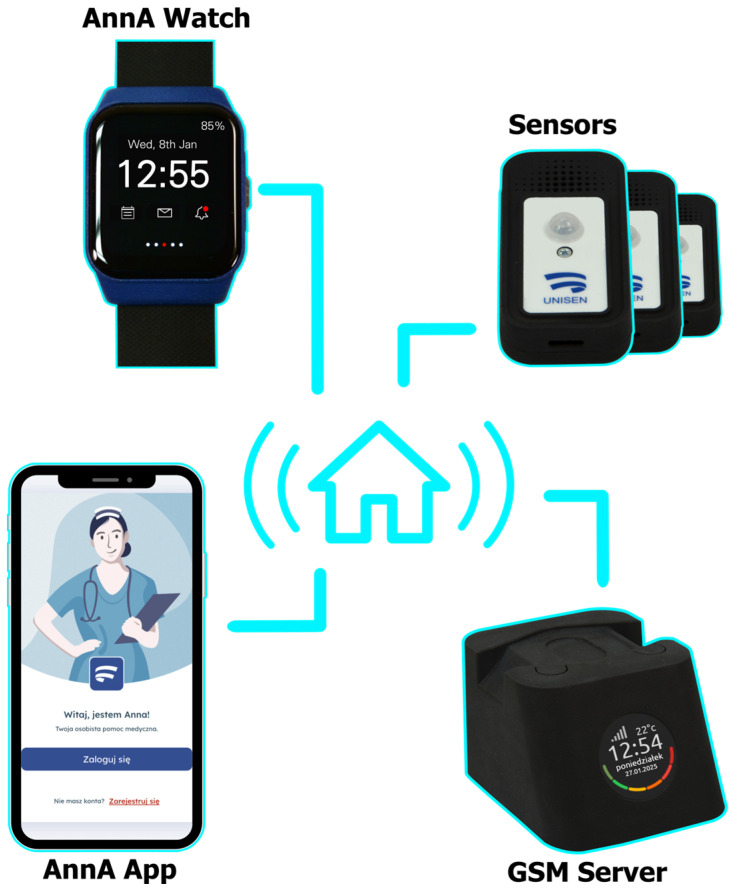
The key elements of Virtual Care Home AnnA.

In response to the demographic challenges posed by an aging society and the growing demand for remote care, the “Virtual Care Home AnnA” project was established. This project was carried out by the company Global R&D in collaboration with the Institute of Electronics at Lodz University of Technology, Poland, under the research project financed by EU The Smart Growth Operational Programme [1]. Its objective is to create an innovative system utilizing electronic wearable devices to enable remote monitoring of the health and well-being of individuals requiring support, while simultaneously preserving their independence in home environment. The system is schematically presented in Figure 1.

The AnnA band advanced smart watch is a key component of this system. This device, equipped with a series of sensors, continuously monitors essential vital parameters, such as body temperature, heart rate, physical activity level (step count and movement), and blood oxygen saturation. A built-in alarm is a crucial feature of the watch, enabling immediate notification of designated individuals in the event of a sudden incident, such as a fall or an abrupt decline in well-being. The use of wireless connectivity is critical for the seamless operation of all the AnnA band watch monitoring and alarm functions, as it allows for the real-time transmission of data to a dedicated AnnA application.

Due to the availability of the unlicensed 2.4 GHz frequency band and the relatively low cost of wireless modules, this band was chosen for the system’s design. The watch communicates with a central module located inside the building via a network of intermediate modules, which form a reliable data transmission infrastructure. This provides caregivers and family members with constant insight into the current health status and activity of the person under care, which significantly enhances the level of safety and effectiveness of remote care.

The continuous development of smartwatch technology has created a growing demand for compact and efficient antennas capable of supporting wireless connectivity in highly constrained environments. Modern smartwatch platforms commonly rely on short-range and wide-area communication standards, including Bluetooth, Wi-Fi, GPS, cellular systems, and, more recently, 5G. In such devices, antenna design is particularly challenging because of the limited available volume, the presence of nearby metallic and electronic components, and the strong electromagnetic interaction between the antenna and the human body. As a result, smartwatch antennas must be designed not only for adequate impedance bandwidth and radiation efficiency, but also for robustness against impedance mismatch, body-induced losses, and compliance with specific absorption rate (SAR) requirements.

A variety of antenna concepts for smartwatch applications has been reported in the literature. For operation in 2.4 GHz band, compact wearable structures with reduced backward radiation and low SAR have been demonstrated, such as the E-shaped antenna with an electromagnetic band-gap structure presented in [2]. Other works have exploited the watch belt or strap as part of the radiating system, showing that antenna performance strongly depends on both the selected topology and its placement, particularly under deformation and in the presence of the user’s wrist [3]. Conformal and multiband concepts integrated into the watch frame, strap, and housing have also been proposed to support several communication standards within a single compact platform [4].

The increasing structural complexity of smartwatch devices has motivated the development of more advanced integration strategies. Metal-bezel antennas, slot antennas, loop antennas, and coupled-feeding techniques have all been investigated as methods for achieving stable performance in compact and metal-rich environments [5,6,7]. In parallel, characteristic mode analysis has become an important design tool for wearable and smartwatch antennas, since it enables a systematic identification of radiating modes with favorable on-body behavior and reduced coupling to biological tissues [8,9,10]. These approaches have been applied not only to sub-6 GHz systems, but also to millimeter-wave smartwatch antennas intended for emerging high-data-rate applications [10,11].

For Wi-Fi and Bluetooth communication in the 2.4 GHz band, antenna miniaturization remains one of the key design issues. Electrically small antennas, circular slot antennas, and flexible strap-integrated antennas have shown that acceptable performance can be achieved despite the strict geometric limitations imposed by smartwatch platforms [12,13,14,15]. Among the compact antenna topologies considered for such applications, the inverted-F family is especially attractive because of its low profile, simple feeding arrangement, compact size, and favorable impedance-matching properties in the vicinity of the human body. In particular, ref. [16] demonstrated the applicability of a PIFA-type solution integrated into the watch strap for 2.4 GHz smartwatch operation.

Despite the significant progress reported so far, the design of smartwatch antennas remains highly dependent on the specific device architecture. The final electromagnetic performance is determined not only by the radiator itself, but also by its interaction with the smartwatch enclosure, display, battery, printed circuit board, and strap, as well as by the intended operating conditions on the user’s wrist. Consequently, each practical implementation requires a dedicated design procedure that accounts for both the mechanical constraints of the device and the electromagnetic influence of the surrounding environment.

Inverted-F and planar inverted-F antennas are widely used in compact wireless devices because of their low profile, simple feeding structure, and relatively easy impedance matching. However, in smartwatch platforms their practical implementation is strongly dependent on the specific device architecture, including the case, ground plane, display, battery, strap, and proximity of the user’s wrist. Therefore, rather than providing a broad review of commercial antenna implementations, the present state-of-the-art analysis is focused on smartwatch and wearable antenna solutions reported in the scientific literature, which are most directly comparable with the proposed design.

Table 1 compares selected smartwatch and wearable antenna solutions reported in the literature with the proposed design. Previous works have investigated several integration strategies, including EBG-backed antennas, metal-belt antennas, conformal radiators, bezel antennas, slot antennas, loop antennas, and strap-integrated structures. In contrast, the originality of the proposed solution lies in the integration of an inverted-F antenna along the side of the smartwatch case, where the available space is strongly limited by the printed circuit board, display, battery, and housing. The radiator length and curvature were adjusted to fit this side region of the case while maintaining operation in the 2.4 GHz ISM band. This geometry also enables simple fabrication from a shaped copper wire, which is advantageous for prototyping and practical implementation in compact wearable devices.

In this context, the present work addresses the design of a compact inverted-F antenna intended for integration into the AnnA band smartwatch and dedicated to Wi-Fi operation in the 2.4 GHz band. The contribution of the paper lies in the development and validation of a side-wall antenna configuration whose radiator length and curvature were adjusted to the available space inside the watch case. The proposed solution was evaluated using a numerical model including the smartwatch structure and the human body, and was further validated by prototype fabrication and measurement. Thus, the paper provides both a practical antenna design for 2.4 GHz smartwatch communication and a structured methodology that may support the development of similar wearable antenna systems.

## 2. Materials and Methods

### 2.1. Antenna Design

As a part of the project, an antenna intended for a smartwatch operating in the widely used 2.4 GHz ISM band was developed. This frequency band is commonly employed by short-range wireless communication standards such as Bluetooth and Wi-Fi, which are essential for the functionality of modern wearable devices. Due to the limited physical dimensions of the smartwatch and the requirement to integrate the antenna with an existing electronic subsystem design, an inverted-F antenna (IFA) topology was selected. Antennas of this type are characterized by their compact size, relatively simple structure, and the capability for straightforward impedance matching to a standard 50 Ω feed. Owing to these advantageous properties, such antennas have been extensively applied in smartwatch and other wearable device designs, as reported in the literature [17,18,19].

For the antenna development purposes, several key design assumptions were adopted. Firstly, the antenna impedance bandwidth was required to fully cover the 2.4 GHz ISM band to ensure reliable operation across all intended communication channels. Secondly, the input impedance of the antenna was assumed to be close to 50 Ω, which is the typical characteristic impedance of radio-frequency transceivers and measurement equipment. Within the operational band, the maximum allowable impedance mismatch was limited such that the voltage standing wave ratio (VSWR) did not exceed a value of 2, ensuring acceptable power transfer efficiency and minimal signal reflections.

Another important design assumption concerned the intended installation environment of the antenna. The antenna was designed for integration into a prototype smartwatch mounted on a prototype printed circuit board with dimensions of 31.2 mm × 37.4 mm. This constraint reflects realistic conditions of wearable electronics, where available space is highly restricted. Furthermore, the antenna dimensions and placement were required to be compatible with other essential components of the device, including the battery and the LCD display. As a result, the antenna design process accounted not only for electromagnetic performance but also for mechanical integration and coexistence with neighboring components, which can significantly influence antenna characteristics in compact wearable systems.

**Figure 2:**
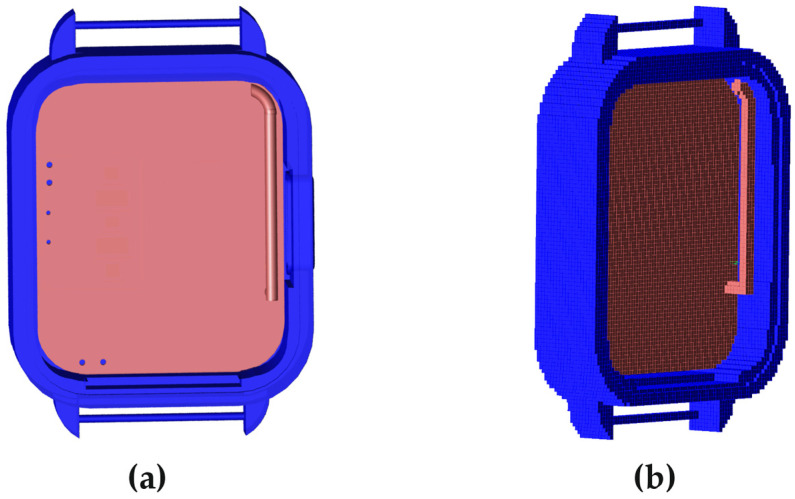
Antenna placed inside the watch case. The battery and LCD screen are removed to show the antenna radiator: (**a**)—element view; (**b**)—mesh view, 0.5 mm voxel size.

Figure 2a presents a general view of the designed inverted-F antenna placed inside the watch case made of ABS material. The radiating element of the antenna was fabricated from a copper wire with a diameter of 1.5 mm and is electrically connected to the ground plane of the printed circuit board. This configuration ensures mechanical robustness of the structure and provides a stable reference potential, which is particularly important in compact wearable devices where vibrations and mechanical stresses may occur during daily use. The overall geometry of the antenna follows the typical F-shaped topology, enabling effective miniaturization while maintaining satisfactory radiation properties in the target frequency band. To represent the small elements that make up the antenna using the FDTD method, local voxel density was applied, reducing their size in the watch region to 0.5 mm. The antenna divided into voxels is shown in Figure 2b.

**Figure 3:**
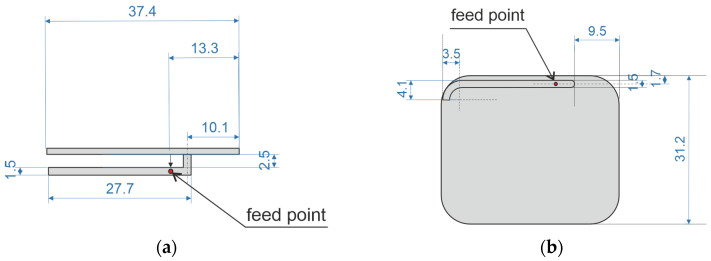
Antenna dimensions: (**a**) top view; (**b**) front view.

The detailed geometrical dimensions of the antenna are illustrated in Figure 3. These dimensions were carefully selected to make optimal use of the available free space inside the smartwatch enclosure. Given the highly constrained internal volume of the device, the antenna layout was adapted to coexist with other components without increasing the overall size of the system. Special attention was paid to the proximity of the antenna radiating element to the smartwatch casing, the battery, and the human body, all of which can significantly influence the electromagnetic behavior of the antenna.

Due to the small distance between the antenna and the surrounding objects (mainly the cover made of ABS), impedance mismatch effects were expected and therefore explicitly considered during the design process. To compensate for this phenomenon, the length of the radiating element was adjusted relative to the initial value calculated for an antenna operating in free space. This correction allowed the antenna to be properly tuned to the desired operating band under realistic conditions, ensuring that the impedance matching requirements were satisfied when the antenna is integrated into the smartwatch and worn on the user’s wrist.

### 2.2. Antenna Model

The antenna modeling and simulation were performed using the Remcom XFdtd (ver. 7.9) commercial electromagnetic software, which is based on the finite-difference time-domain (FDTD) method, in conjunction with a numerical model of the human body [20,21]. The FDTD technique enables full-wave analysis of complex electromagnetic structures in the time domain and is particularly well suited for evaluating antenna performance in the presence of heterogeneous and lossy media, such as biological tissues. In the adopted human body model, the computational domain was discretized into cubic voxels with an edge length of 2 mm, providing a compromise between numerical accuracy and computational efficiency.

The simulations were performed for a model with 2 mm voxels, reduced to 0.5 mm voxels in the watch region. The model uses 163,773,144 voxels and occupies 5.2 GB of RAM. A boundary condition corresponding to free space (Perfectly Matched Layer) was applied.

The numerical human body model used in this study includes 39 tissue types with frequency-dependent dielectric properties. Although more anatomically detailed models, such as the Virtual Population models [22], are available in the literature, the adopted model was considered sufficient for the present antenna-design study because the region of interest was limited mainly to the wrist and forearm. In this region, the dominant tissues affecting antenna performance are skin, fat, muscle, bone, and blood vessels, which are represented in the adopted model. Nevertheless, the use of a model with a limited number of tissue classes is acknowledged as a limitation of the study, especially for more detailed SAR or anatomical variability analyses.

**Figure 4:**
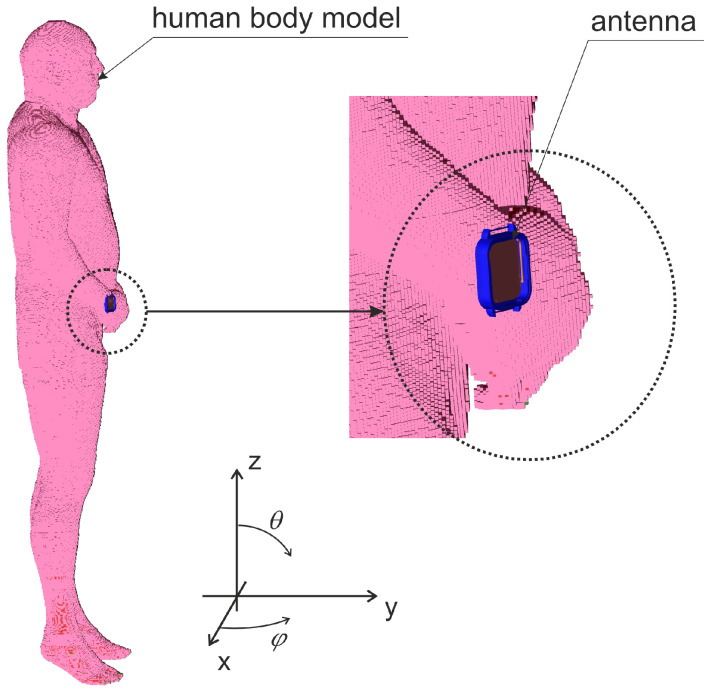
Numerical model of the antenna integrated into the prototype smartwatch, positioned on the wrist of the right hand without the strap.

Figure 4 illustrates the numerical model of the antenna integrated into the prototype smartwatch, positioned on the wrist of the right hand. This configuration reflects a typical usage scenario and was selected to capture the most relevant electromagnetic interactions between the antenna and the surrounding tissues.

**Figure 5:**
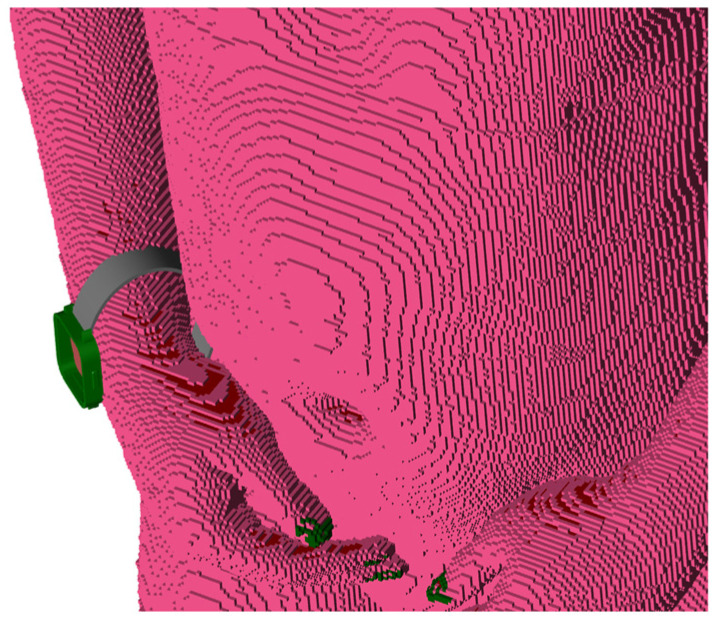
Numerical model of the antenna integrated into the prototype smartwatch, positioned on the wrist of the right hand with the model of textile strap.

To investigate the effect of the wrist strap on the antenna parameters, a numerical model with a strap was developed. The strap was assumed to be made of a 2 mm thick textile material with a permittivity of 1.5 and a dielectric loss angle of 0.01, corresponding to a synthetic knit fabric [23]. The strap model is shown in Figure 5.

The numerical model employed in the simulations included the antenna radiating element, the printed circuit board, the housing fabricated from an ABS material, as well as the LCD display and the battery. In order to accurately reproduce the internal structure of the device, the LCD display and the battery were modeled together with a metallic foil layer surrounding them on the inner side of the enclosure. This conductive layer, which is commonly used in practical devices for shielding and structural purposes, can significantly influence the electromagnetic field distribution and, consequently, the antenna impedance characteristics. Therefore, its inclusion in the numerical model was necessary to ensure realistic simulation results.

The material properties used in the numerical model are summarized in Table 2. Metallic elements, including the antenna radiator, PCB ground plane, and shielding layers of the battery/display model, were represented as perfect electric conductors. Dielectric elements were modeled using the relative permittivity and loss parameters listed in the table. The human body was represented by a heterogeneous model containing 39 tissue types with frequency-dependent dielectric properties.

## 3. Results

### 3.1. Simulations Results

**Figure 6:**
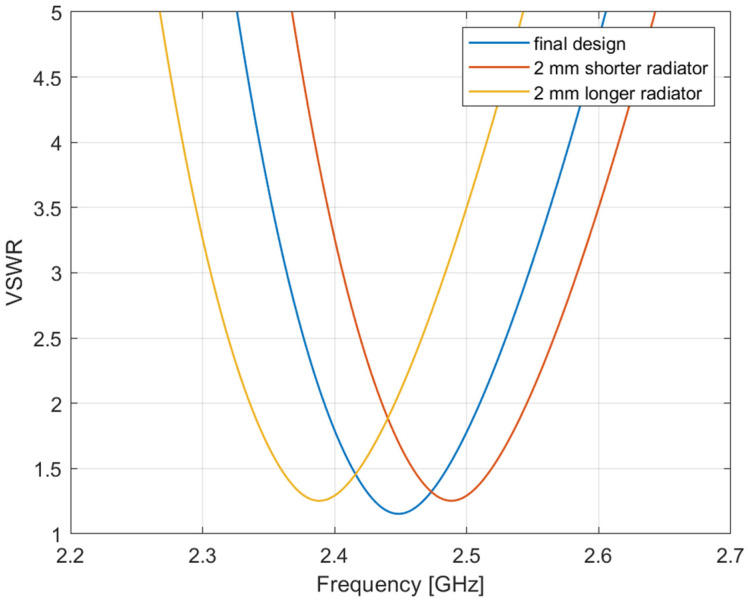
Influence of the radiator length on the simulated VSWR characteristics.

To clarify the antenna tuning procedure, a parametric analysis of the radiator length was performer with step-by-step simulations. Figure 6 shows the simulated VSWR characteristics for the final antenna geometry and for two modified variants, in which the radiator was shortened and lengthened by 2 mm. The results confirm that the radiator length is the main parameter controlling the resonant frequency. Shortening the radiator shifts the resonance toward higher frequencies, whereas increasing its length shifts the resonance toward lower frequencies. The final radiator length was selected so that the best impedance matching was obtained within the 2.4 GHz ISM band.

**Figure 7:**
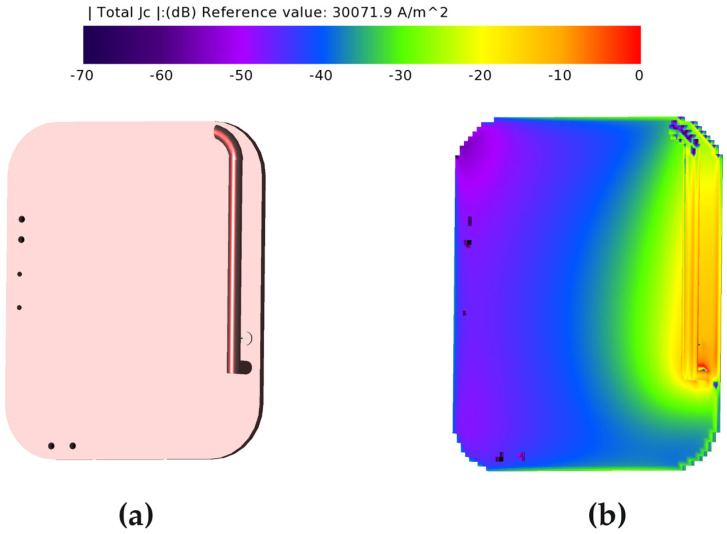
Current distribution on antenna radiator and ground plane: (**a**)—radiator; (**b**)—current density.

Figure 7 Presents the results of current distribution simulation for the frequency of 2.45 GHz obtained for antenna F-shaped radiator and antenna ground plane located beneath, for the final design.

**Figure 8:**
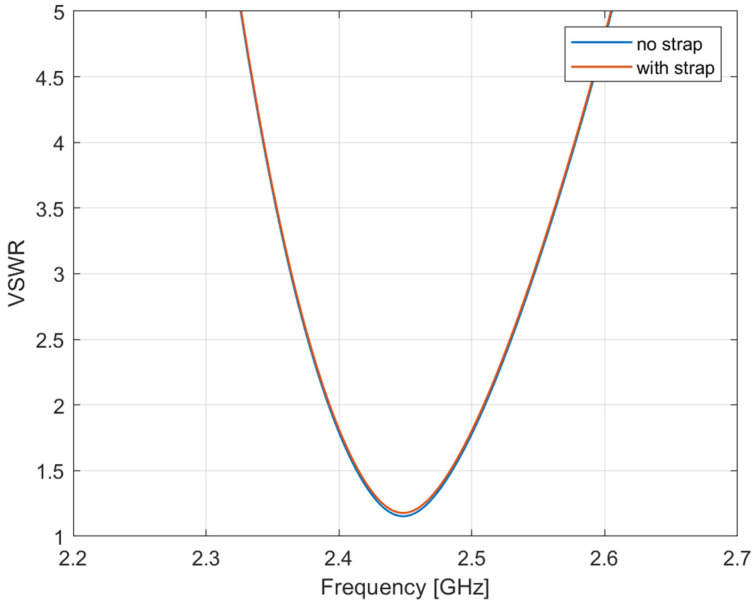
Results of antenna impedance matching obtained from simulations with and without wrist strap.

Computer simulations of the antenna impedance matching were carried out for the smartwatch placed on the human arm, representing typical operating conditions during normal use. This configuration allowed the combined effects of the device components and the proximity of the human body to be considered. The simulation results obtained for the numerical model, as presented in Figure 8, indicate satisfactory impedance matching of the antenna within the assumed operating frequency band. Particularly good matching performance was achieved over the frequency range from 2.4 GHz to 2.5 GHz, confirming that the design requirements defined for operation in the 2.4 GHz ISM band were met under realistic usage conditions, with VSWR below 1.8. The results obtained for antenna model with and without a wristband are almost identical.

Using the developed numerical model, simulations of the antenna radiation pattern were obtained for the antenna integrated into the device and positioned on the right wrist of the human body. For the purposes of these simulations, a free-space boundary condition was applied to eliminate the influence of finite ground or substrate on the obtained results. This approach is equivalent to the conditions of an anechoic chamber, in which subsequent experimental verification measurements were performed, and therefore ensures consistency between numerical and measurement environments.

**Figure 9:**
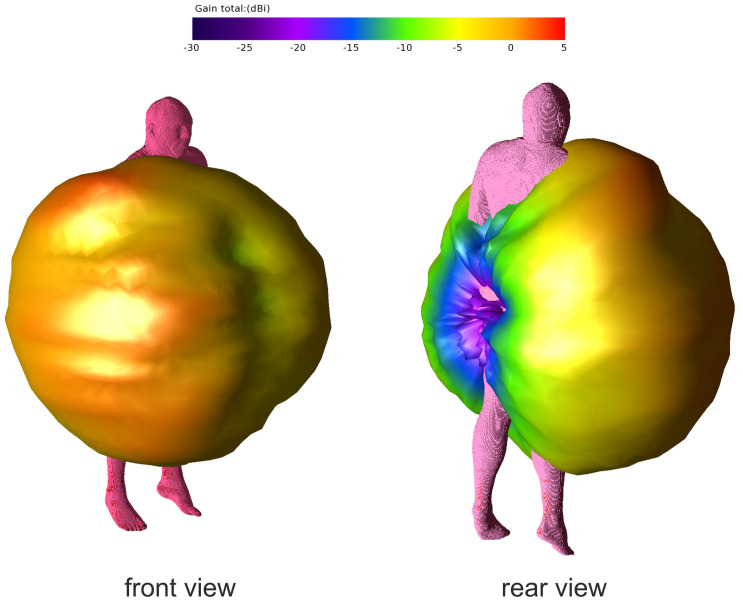
Results of radiation pattern simulations in 3D obtained from simulations.

**Figure 10:**
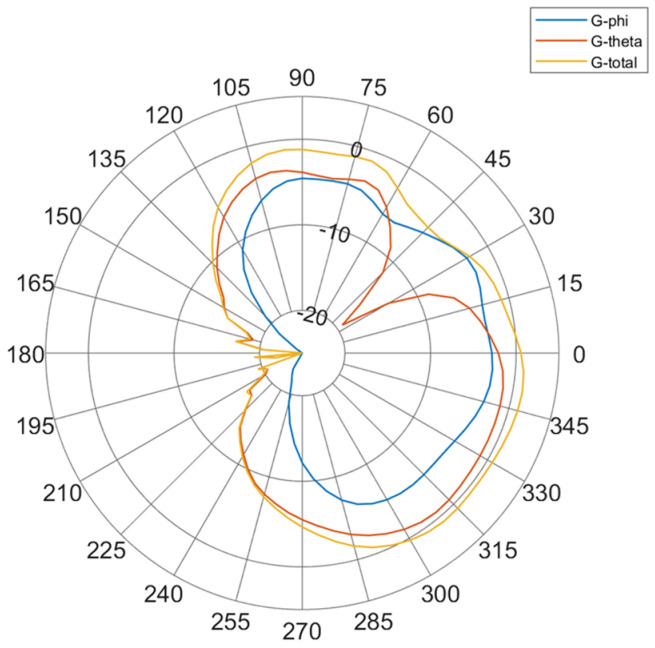
Results of radiation pattern simulations in the horizontal plane.

The simulated radiation pattern in 3 dimensions is presented in Figure 9 while the radiation pattern in the horizontal x-y plane is shown in Figure 10. The results are expressed in terms of antenna gain. For the antenna placed on the human body, the maximum gain was equal to −0.7 dBi for vertical polarization (*G_theta_*) and −2.6 dBi for horizontal polarization (*G_phi_*). The value of the total simulated gain reached 1.1 dBi.

Based on the simulation results shown in Figure 9, a pronounced impact of the human body on the antenna radiation pattern can be observed. The absorption and dissipation of electromagnetic energy within biological tissues lead to significant attenuation of the radiated power, particularly in directions that are partially or fully obstructed by the body. As a consequence, the antenna gain is noticeably reduced in these directions, which is a typical and unavoidable effect in wearable antenna applications operating near the human body.

**Figure 11:**
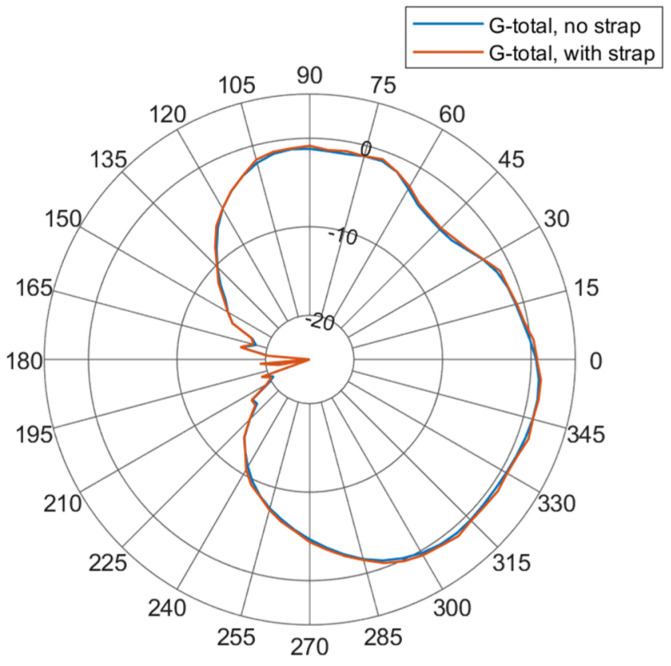
Results of radiation pattern simulations in horizontal plane, total gain obtained for model with and without a wrist strap.

To verify the potential impact of the watch strap on the radiation characteristics, radiation characteristics were simulated for the model with and without the strap. The results showing the total gain for the x-y plane, shown in Figure 11, are very similar. This indicates that the strap has a negligible effect on the antenna radiation.

**Figure 12:**
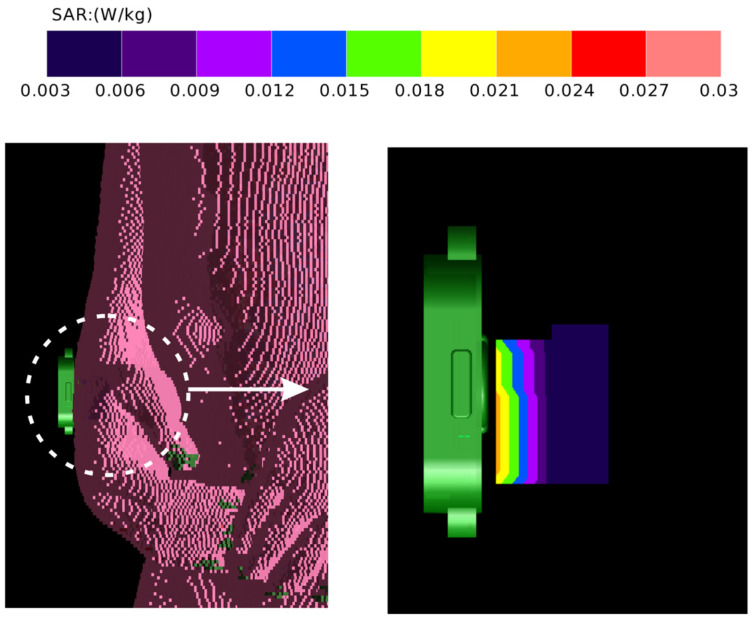
Results of SAR simulations averaged for 1 g mass of tissue. The white dashed circle indicates the enlarged area for which the SAR distribution is presented.

The performance of the designed antenna was also verified in terms of electromagnetic energy absorption in the human body. For this purpose, SAR simulations were performed in XFdtd using the smartwatch antenna placed on the wrist of the heterogeneous human body model. The results were normalized to an input power of 100 mW, corresponding to the assumed maximum transmitter power in the 2.4 GHz band. The maximum SAR averaged over 10 g of tissue was 0.55 W/kg, whereas the maximum SAR averaged over 1 g of tissue was 1.5 W/kg. The maximum SAR occurred in the wrist region closest to the smartwatch antenna. Despite assuming the maximum transmitter power, the obtained values were significantly below the European exposure limit for limb exposure, equal to 4 W/kg averaged over 10 g of tissue [22]. The SAR distribution averaged over 1 g of tissue is presented in Figure 12. The relatively low SAR values result from the fact that neither the antenna radiator nor the antenna ground plane is in direct contact with the skin, as both are separated from the body by the dielectric cover of the smartwatch.

### 3.2. Measurements Results

**Figure 13:**
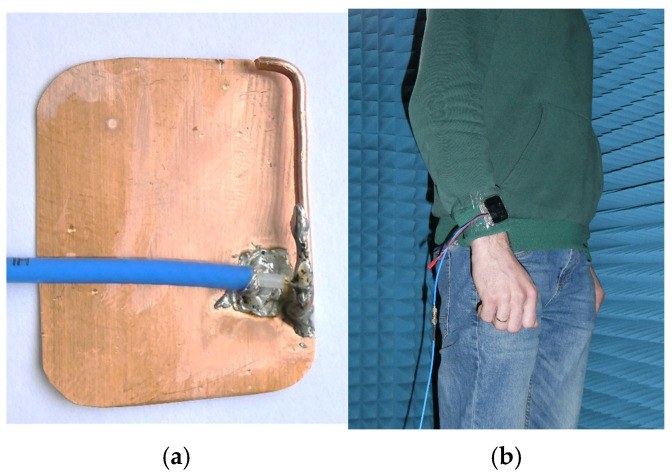
The prototype antenna: (**a**) removed from the smartwatch; (**b**) placed on human subject for measurements.

The developed antenna design was experimentally verified by means of measurements carried out using a prototype antenna, which is shown in Figure 13. In the prototype, the ground plane of the printed circuit board, to which the radiating element is connected, was represented by an element fabricated from a copper sheet with a thickness of 0.5 mm and shape of the mentioned PCB. The antenna radiating element itself was manufactured from a copper wire with a diameter of 1.5 mm, ensuring good electrical conductivity and mechanical stability of the structure. The antenna was fed by a Huber + Suhner Multiflex 86 coaxial cable terminated with an SMA connector, which is commonly used in radio-frequency measurements due to its well-defined characteristic impedance and repeatable performance.

The experimental verification of the antenna parameters was conducted under anechoic chamber conditions in order to minimize the influence of external electromagnetic interference and unwanted reflections. The antenna was integrated into a prototype device, which was then attached to the wrist of a human subject, as illustrated in Figure 13. This measurement configuration closely reflects realistic operating conditions of a smartwatch and allows the combined effects of the device enclosure and the human body on the antenna performance to be assessed.

It should be noted that the antenna itself is not intended to be in direct electrical or mechanical contact with the skin. In the smartwatch, direct skin contact is required only for the optical pulse-oximeter sensor, which is located on the back side of the device. The antenna and its ground plane are integrated inside the watch case and are separated from the user’s body by the dielectric cover of the smartwatch. Therefore, the presence of a thin textile layer between the arm and the device during the radiation-pattern measurements was not expected to significantly affect the antenna performance. This assumption is also consistent with the numerical comparison of models with and without the textile strap, which showed only minor differences in VSWR and radiation pattern.

**Figure 14:**
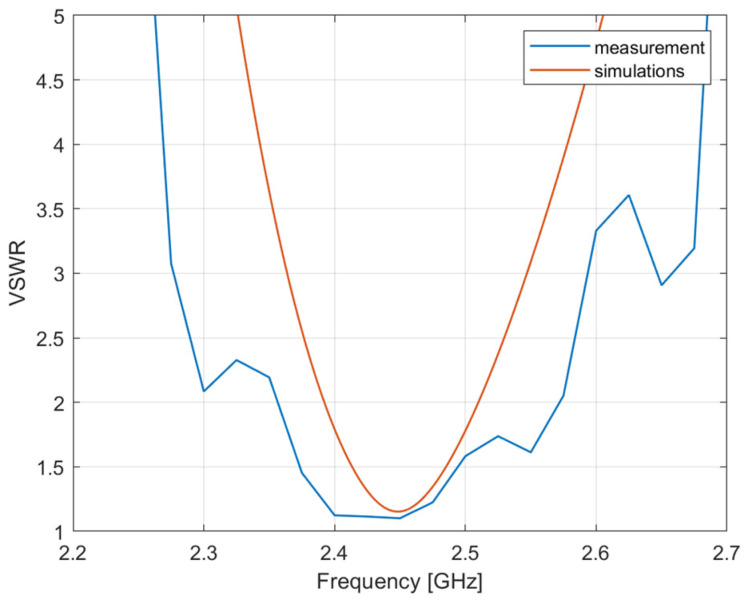
The results of antenna impedance matching measurements (blue) and simulations (red).

Impedance matching measurements were performed using an MS4647B vector network analyzer fabricated by Anritsu (Morgan Hill, CA, USA). The calibration reference plane was shifted to the opening of the coaxial cable, thereby eliminating the influence of the feeding line on the measured results and ensuring accurate characterization of the antenna input impedance. The measured impedance matching with respect to the reference impedance $Z_{0}=50\;\Omega$ was presented in the form of the voltage standing wave ratio (VSWR), as shown in Figure 14. The obtained results provide a direct basis for evaluating the conformity of the prototype antenna with the design assumptions and the numerical simulation outcomes. The antenna impedance matching is presented in Figure 14 in terms of VSWR. This form was selected because the antenna bandwidth requirement in this work was defined for VSWR < 2. It should be noted that VSWR contains the same impedance-matching information as the reflection coefficient S11, since both quantities are directly related through the magnitude of the reflection coefficient. In particular, VSWR < 2 corresponds to |S11| < −9.54 dB.

The overall radiation performance of the antenna was evaluated numerically using the simulated three-dimensional gain pattern shown in Figure 9. The measured radiation characteristics were presented as two-dimensional cuts in the horizontal x-y plane, which is the most relevant plane for off-body communication in the considered indoor monitoring scenario. Full three-dimensional radiation-pattern measurements were not performed because the available measurement setup used a turntable rotating around the vertical axis, which enables reliable acquisition of azimuthal pattern cuts but not a complete 3D pattern. Moreover, for wearable antennas, the radiation pattern strongly depends on the body position, antenna orientation, and effective ground-plane configuration; therefore, comparison of representative pattern cuts is a commonly used practical validation approach.

Prototype verification also included radiation-pattern measurements of the device operating under the same on-body conditions as described above. The measurements were performed in an anechoic chamber at a distance of 4 m between the antenna under test and the measurement antenna, which satisfied the far-field condition for the considered antenna size at 2.45 GHz. The measurement setup consisted of a SMA100 signal generator, a FSC6 spectrum analyzer, and HF-907 measurement antennas fabricated by Rohde & Schwarz (Memmingen, Germany). During the measurements, the human subject wearing the smartwatch prototype was placed on a turntable rotating around the vertical axis. Therefore, the radiation pattern was measured in the horizontal x-y plane, which is the most relevant plane for off-body communication in the considered indoor monitoring scenario.

**Figure 15:**
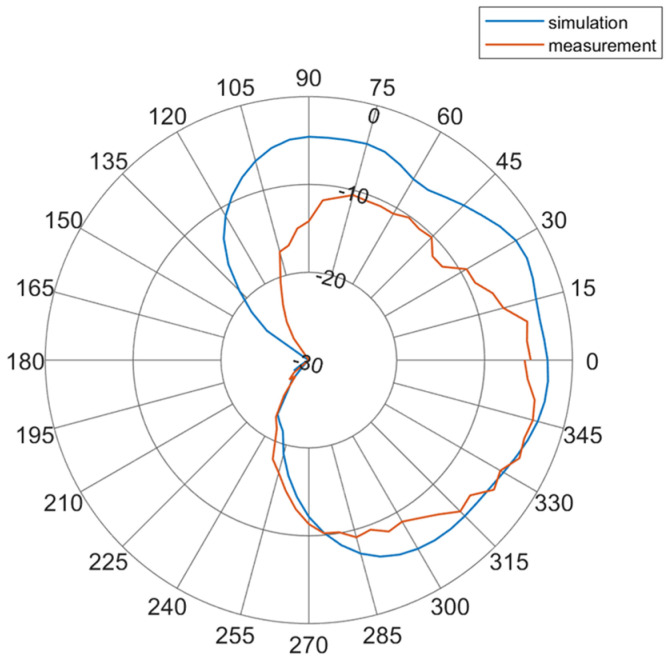
Simulations and measurement results of antenna gain for linear horizontal polarization: *G_phi_*.

**Figure 16:**
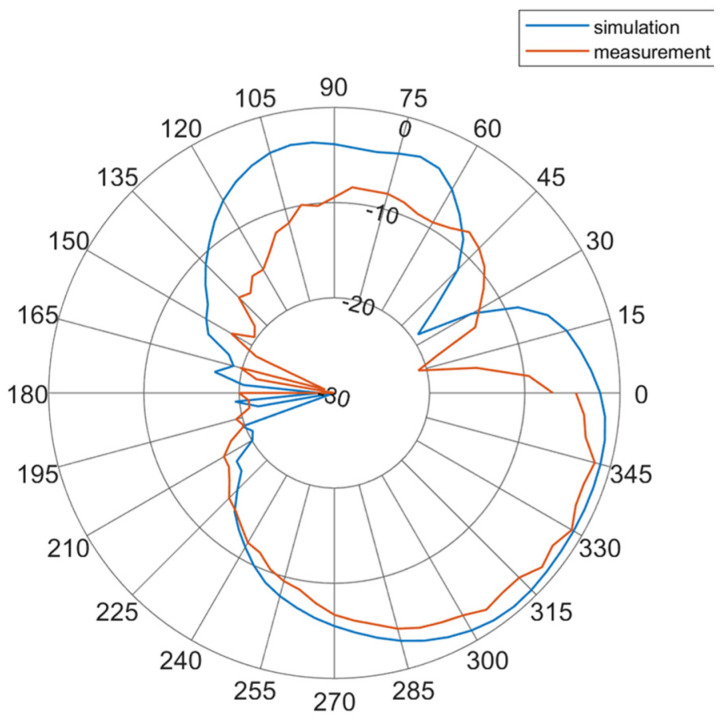
Simulations and measurement results of antenna gain for linear vertical polarization: *G_theta_*.

The antenna gain was estimated using a reference-antenna method. The HF-907 antenna fabricated by Rohde & Schwarz (Memmingen, Germany) was first used as a calibrated reference antenna, and the received power level was then compared with the level obtained for the smartwatch antenna under the same measurement conditions. The feeding cable was routed and fixed so as to minimize its influence on the measured pattern. The impedance measurements were calibrated at the end of the coaxial cable using open- and short-circuit standards. The obtained radiation-pattern results are presented in Figure 15 for horizontal polarization, *G_phi_*, and in Figure 16 for vertical polarization, *G_theta_*. The maximum measured gain was −3.9 dBi for horizontal polarization and −1 dBi for vertical polarization.

Experimental SAR measurements were not performed because they require a dedicated standardized SAR measurement facility with tissue-equivalent phantoms and calibrated electric-field probes. Therefore, SAR was evaluated numerically using the FDTD method and a heterogeneous human body model, which is a standard approach at the antenna-design stage.

### 3.3. Simulations of Antenna Performance in the Indoor Environment

**Figure 17:**
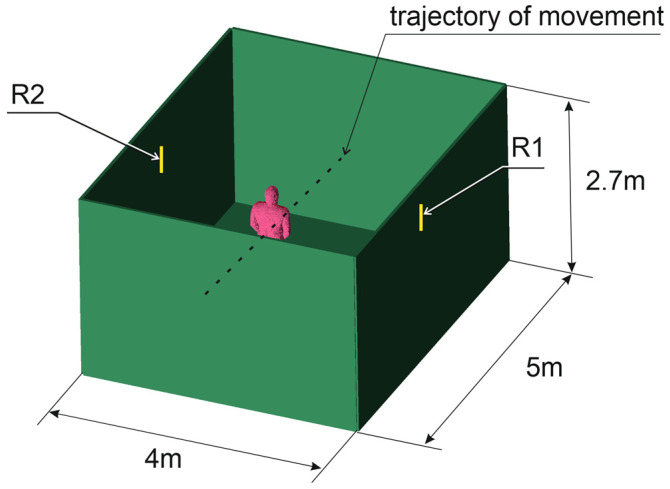
Model of the indoor system setup. The dashed line indicates the person’s movement trajectory.

To verify the antenna performance when deployed in an indoor human monitoring system, simulations were carried out for a setup consisting of a transmitter embedded in a smartwatch and two receivers placed within the room. The system considered for this purpose is shown in Figure 17, where the ceiling has been hidden for clarity. The room dimensions were 4 m × 5 m × 2.7 m. The walls were assumed to be made of concrete with a relative permittivity of 3.4 and a dielectric loss tangent of 0.05 [22].

The receivers were mounted on opposite walls at a height of 2 m above the floor, in the middle of the room. Both receivers were equipped with half-wave dipole antennas designed for 2.45 GHz, which was also the operating frequency used in the simulations. Receiver R1, shown in the figure, was located on the same side as the smartwatch worn on the subject’s right wrist. Receiver R2 was positioned on the opposite side of the room, in the region where radiation was shadowed by the human body.

The indoor propagation simulations were performed using Remcom XFdtd software (ver. 7.9), based on the finite-difference time-domain (FDTD) method. The computational model used a nonuniform voxel resolution: 10 mm voxels were applied in the room region, the resolution was refined to 2 mm in the human-body region, and further refined to 0.5 mm in the smartwatch region. The complete numerical model consisted of 725,271,690 voxels and required approximately 22 GB of RAM. A free-space boundary condition was applied using perfectly matched layers (PMLs).

**Figure 18:**
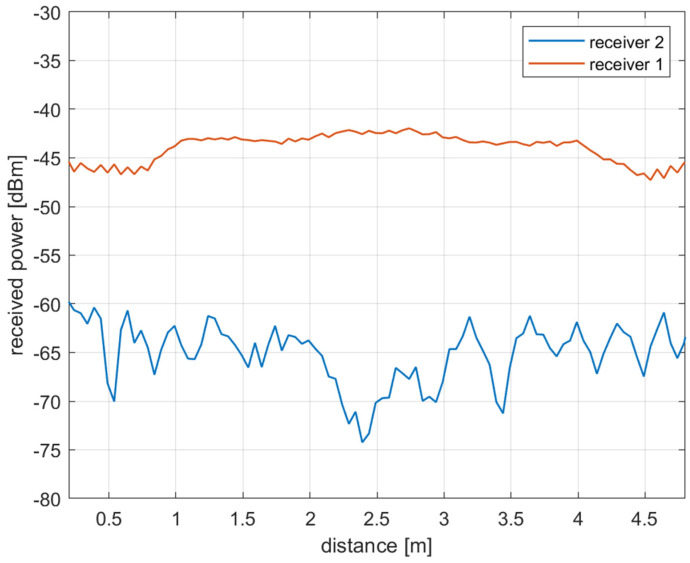
Power received at receiver R1 and receiver R2 for different transmitter locations.

The received power at both receivers was evaluated for a person moving along a path parallel to the longer wall, as indicated in Figure 16. The simulations were performed for consecutive transmitter displacements of 5 cm, assuming a smartwatch transmitter power of 0 dBm. The obtained results are presented in Figure 18.

The median received power was −43 dBm for receiver R1 and −64 dBm for receiver R2. In both cases, the received power remained well above the assumed receiver sensitivity of −100 dBm, indicating a considerable link-budget margin for the analyzed indoor configuration.

The purpose of the indoor simulation was not to provide a comprehensive analysis of indoor propagation, but rather to give an additional system-level indication of the antenna performance in a representative monitoring scenario. The receiver sensitivity of −100 dBm was adopted as a reference threshold for low-power 2.4 GHz wireless communication. Both receivers were modeled as half-wave dipole antennas operating at 2.45 GHz, with fixed orientation during the simulation. Consequently, the effects of polarization, antenna orientation, human-body shadowing, wall reflections, and room geometry were included in the full-wave FDTD analysis for the considered configuration. Therefore, the obtained received-power values should be interpreted as an example of the achievable link margin in the analyzed room setup, rather than as a general indoor propagation model.

## 4. Discussion

The obtained results confirm that the proposed smartwatch antenna satisfies the assumed design requirements and is suitable for operation in the 2.4 GHz ISM band. In particular, the measured impedance matching demonstrates robust performance, with a minimum VSWR of 1.1 and an operational bandwidth of 2.36–2.57 GHz for VSWR < 2. The simulated bandwidth for the same criterion was 2.39–2.51 GHz. Thus, the measured lower band edge was shifted downward by approximately 30 MHz, whereas the upper band edge was shifted upward by approximately 60 MHz. Although the measured bandwidth is slightly wider than that predicted by simulation, both results fully cover the intended operating band. The observed differences may result from fabrication tolerances, small deviations in the radiator geometry, uncertainty in the dielectric parameters of the smartwatch case, simplified modelling of internal components, and the influence of the feeding cable and measurement configuration. From an application point of view, the wider measured bandwidth is advantageous, as it suggests that the fabricated antenna is relatively tolerant to manufacturing deviations and environmental influences. This feature is particularly important in wearable systems, where the proximity of the human body may alter the antenna input impedance and shift the resonant frequency [23,24,25].

The radiation patterns are also consistent with the expected behavior of a wearable antenna operating in close proximity to the human body. The observed shift in the maximum gain direction above the x-y plane for the antenna placed vertically on the human arm can be attributed to the influence of the lossy biological tissues, which partially absorb and distort the electromagnetic field [23,24]. Such behavior has been widely reported in previous studies on body-worn antennas and is regarded as a typical consequence of on-body deployment [25,26]. Therefore, the measured radiation pattern may be considered satisfactory from the perspective of practical smartwatch operation.

For wearable communication systems, the antenna performance in the horizontal plane is of particular importance, since this plane is closely related to off-body communication in indoor environments. In the present case, the dominant gain component in the x-y plane is the vertically polarized component $G_{theta}$, which indicates that the antenna preserves the intended main polarization. However, the cross-polarized component $G_{phi}$ is also relatively strong. This can be explained by the curved termination of the radiator, which introduces additional current paths and consequently increases the cross-polarized field component. Although a high cross-polarization level may be regarded as a departure from ideal linear polarization, in practical indoor wearable links it may also be beneficial, since it can reduce polarization mismatch under varying arm orientations and multipath propagation conditions [26,27,28]. Thus, the obtained polarization characteristics should be interpreted as a compromise between compact geometry and polarization purity. The maximum values of antenna gain for the x-y plane are presented in Table 3.

SAR simulations in XFdtd confirmed that the proposed smartwatch antenna meets human-exposure safety requirements. At 100 mW transmit power in the 2.4 GHz band, the obtained SAR values remained well below the European limit, confirming the antenna’s suitability for safe wearable operation.

A comparison between simulation and measurement shows very good agreement in terms of the overall radiation pattern shape, which confirms the correctness of the adopted numerical model. At the same time, the measured maximum gain values are lower than those obtained in simulation. The most likely explanation is the discrepancy between the electromagnetic parameters assumed for the watch casing materials in the model and the actual parameters of the fabricated prototype. In wearable antenna design, even small inaccuracies in dielectric constant, loss tangent, geometry, or spacing between antenna and body may noticeably influence the gain and efficiency [24,27]. Therefore, the observed difference should not be interpreted as a failure of the design, but rather as a typical consequence of the simplified assumptions required in numerical modeling. Considering the very small dimensions of the antenna and the constraints imposed by smartwatch integration, the measured gain values remain acceptable for the intended short-range wireless application [24,29].

The system-level simulations performed for the indoor human-monitoring scenario provide additional confirmation of the practical usefulness of the proposed antenna. The median received power values of −43 dBm for receiver R1 and −64 dBm for receiver R2 are substantially higher than the assumed receiver sensitivity of −100 dBm. This corresponds to large link-budget margins, indicating that reliable communication can be maintained even under less favorable propagation conditions.

The difference observed between the two receiver locations is also significant, as it reflects the sensitivity of off-body propagation to room geometry, human-body shadowing, and antenna orientation [28,29,30]. Receiver R1 was located on the same side as the smartwatch worn on the right wrist, whereas receiver R2 was positioned on the opposite side, where the signal was additionally affected by body shadowing. If the positions of the receivers were changed, the received power values would also change depending on line-of-sight obstruction, antenna orientation, distance to the transmitter, and multipath conditions in the room [28,30]. In general, a receiver located on the same side as the smartwatch is expected to obtain a higher received power, while a receiver located behind the body or in a shadowed region is expected to receive a weaker signal.

Previous studies on indoor body-centric channels have shown that the received signal level may vary considerably depending on the position of the receiving node and the surrounding multipath environment [28,30]. Therefore, the presented results should not be interpreted as universal received-power values for all indoor configurations, but rather as an example showing the expected difference between favorable and less favorable receiver locations. Since both receivers still provided a large margin above the assumed receiver sensitivity, the results indicate that the proposed antenna provides sufficient robustness for practical indoor monitoring applications.

From a broader perspective, the presented findings demonstrate that the proposed antenna design offers a favorable balance between compact size, satisfactory impedance matching, acceptable radiation performance, and sufficient link margin for wearable operation. This is an important outcome, since smartwatch antennas are subject to severe space limitations and must operate efficiently in the presence of a strongly perturbing nearby body environment [23,25,29]. The results therefore support the initial hypothesis that an antenna integrated into a smartwatch can provide reliable wireless connectivity for human-monitoring systems without requiring excessive dimensions or complex structures.

Nevertheless, some limitations should be acknowledged. The experimental validation was performed for a specific prototype and a specific wearing configuration, whereas practical usage conditions may vary considerably depending on the user, wrist size, arm posture, strap properties, and surrounding environment. These factors may influence both the matching and radiation characteristics, as well as the channel statistics [24,28]. For this reason, future work should include measurements on a larger group of users and under more diversified operating conditions. It would also be beneficial to investigate the sensitivity of antenna performance to the dielectric properties of the watch housing and other integration materials. Furthermore, additional experimental studies in realistic indoor environments, including packet error rate, RSSI fluctuations, and dynamic body movements, would provide a more complete assessment of the antenna performance at the system level.

Normal human activity may influence the antenna performance, mainly through changes in arm position, body shadowing, antenna orientation, and polarization mismatch. In particular, arm movement can modify the radiation pattern because the attenuation introduced by the human body depends on the relative position of the antenna and the body. A detailed analysis of dynamic activity scenarios would require time-consuming simulations or measurements for multiple body postures and motion conditions. Therefore, such analysis was beyond the scope of the present work and is planned as future research.

Overall, the results indicate that the proposed smartwatch antenna is a promising solution for compact wearable devices operating in indoor human-monitoring systems. The good agreement between simulation and measurement, together with the favorable link-budget results, confirms the validity of the design approach and suggests that further optimization may focus primarily on improving stability and polarization control rather than on basic impedance matching performance.

To quantitatively compare the proposed antenna with selected smartwatch and wearable antenna solutions, Table 4 summarizes key parameters reported in the literature. It should be noted that the bandwidth criteria are not identical in all works; therefore, the comparison should be treated as indicative rather than strictly one-to-one.

Compared with the selected works, the proposed antenna provides moderate gain, which is expected due to its compact size, side-wall placement, proximity to conductive smartwatch components, and operation close to the human body. However, it fully covers the 2.4 GHz ISM band with VSWR < 2, shows SAR values below the applicable exposure limits, and is validated by both numerical body modelling and prototype measurements. The main advantage of the proposed solution is therefore not the highest gain or efficiency, but the practical integration of a curved wire inverted-F radiator into the constrained side region of a specific smartwatch prototype.

## 5. Conclusions

This paper presented the design, numerical analysis, and experimental validation of a compact inverted-F antenna intended for integration into the AnnA smartwatch operating in the 2.4 GHz ISM band. The antenna was designed under strong geometrical constraints imposed by the smartwatch structure, including the printed circuit board, display, battery, housing, and proximity of the user’s body. The influence of the human body was considered using full-wave FDTD simulations with a heterogeneous body model. In addition, the antenna performance was investigated in a representative indoor human-monitoring scenario to provide a system-level indication of the achievable link margin.

The prototype measurements confirmed satisfactory impedance matching in the intended operating band. The measured VSWR did not exceed 1.5 across the 2.4 GHz ISM band, indicating good matching between the antenna and the transmission system. The measured radiation-pattern cuts showed a similar overall shape to the simulated results. The maximum measured gain was −1 dBi for vertical polarization, which was lower than the simulated value. This difference may result from discrepancies between the material parameters assumed in the numerical model and the actual properties of the smartwatch case, as well as from fabrication tolerances, cable influence, and simplified modelling of internal components. Nevertheless, the measured gain level can be considered acceptable for the analyzed short-range wearable communication scenario, especially considering the miniature antenna size and the presence of nearby conductive and dielectric components.

The SAR analysis showed that the obtained values were below the applicable exposure limits for the assumed transmitter power. The indoor simulation results also indicated a large link-budget margin for the considered room configuration; however, these results should be interpreted as representative for the analyzed scenario rather than as a general indoor propagation model.

Overall, the obtained results confirm the feasibility of the proposed antenna for the considered AnnA smartwatch prototype and measurement configuration. The conclusions are limited to the specific prototype geometry, one main wearing configuration, and static measurement conditions investigated in this study. Future work should include additional measurements for different users, wrist positions, arm postures, and dynamic activity scenarios. Further research will also focus on verifying the possible use of flexible thin-film laminate for antenna fabrication, which may require modifications of the smartwatch case but could simplify large-scale production of the AnnA band device.

## Figures and Tables

**Table 1:** Comparison of selected smartwatch and wearable antenna solutions with the proposed design.

Ref.	Antenna Type	Band/Application	Integration Approach	Main Distinction
[2]	E-shaped antenna with EBG	2.4 GHz smartwatch	EBG-backed wearable radiator	Low SAR and reduced backward radiation
[3]	Metal-belt antenna	Cellular smartwatch	Watch belt used as radiator	Belt-based antenna integration
[4]	Conformal antenna	Multiband smartwatch	Frame/strap/housing integration	Conformal multiband concept
[5]	Metal-bezel antenna	Dual-band smartwatch	Coupled-fed metal bezel	Stable operation in metal-rich case
[6]	Slot antenna	All-metal smartwatch	Slot in metallic structure	Suitable for all-metal casing
[7]	Loop antenna	Smartwatch	Integrated loop structure	Compact loop-type radiator
[8]	CMA-based antenna	Tri-band smartwatch	Mode-based smartwatch design	Low SAR and high on-body efficiency
[9]	Full-screen antenna	Multiband IoT smartwatch	Full-screen integration	Compact multiband IoT design
[10]	mm-wave antenna	Smartwatch/IoT	Compact mm-wave radiator	Link-budget analysis included
[11]	mm-wave wearable antenna	5G smartwatch	Smartwatch-integrated antenna	High-frequency 5G operation
[12]	Electrically small antenna	Smartwatch dial	Dial-integrated radiator	Miniaturized tunable structure
[13]	Strap antenna	Wideband smartwatch	Strap-integrated radiator	Circular polarization
[14]	Compact smartwatch antenna	Bluetooth/5G	Smartwatch platform integration	Dual-service operation
[15]	Flexible strap antenna	Wi-Fi/Bluetooth	Flexible strap radiator	Flexible wearable implementation
[16]	PIFA	2.4 GHz smartwatch	Strap-integrated PIFA	Closest 2.4 GHz PIFA solution
This work	Wire inverted-F antenna	2.4 GHz AnnA smartwatch	Side of watch case; curved wire radiator	Adjusted length and curvature enabling compact wire fabrication

EBG—electromagnetic band gap; CMA—characteristic mode analysis; PIFA—planar inverted-F antenna.

**Table 2:** Material properties used in the numerical model.

Component	Material Model	Relative Permittivity	Loss Tangent/Conductivity	Comment
Antenna radiator	Copper/PEC	—	PEC	1.5 mm wire radiator
PCB ground plane	Copper/PEC	—	PEC	Ground reference for the antenna
PCB substrate	FR-4/dielectric	4.3	0.025	Used for the smartwatch PCB
Watch case	ABS	3.1	0.011	Dielectric housing surrounding the antenna
Battery/display shielding	Metallic layer/PEC	—	PEC	Conductive layers included in the model
Wrist strap	Synthetic textile	1.5	tanδ = 0.01	2 mm thick textile layer [23]
Human body	Heterogeneous tissue model	Frequency-dependent	Frequency-dependent	39 tissue types

**Table 3:** Antenna maximum gain in x-y plane, obtained from measurements and simulations.

Gain Component	Simulations [dBi]	Measurement [dBi]
*G_theta_*	−0.7	−1
*G_phi_*	−2.6	−3.9
*G_total_*	1.1	x

**Table 4:** Comparison of key antenna parameters reported for selected smartwatch and wearable antenna solutions.

Ref.	Antenna Type	Band	Gain	VSWR	Dimensions
[2]	E-shaped antenna with EBG	2.4 GHz	4.9 dBi	<2	30 × 20 × 0.75 mm^3^
[3]	Metal-belt antenna -dipole	700 MHz 2.7 GHz	NR	<3.5	145 × 40 × 2 mm^3^
[3]	Metal-belt antenna -monopole1	700 MHz 2.7 GHz	NR	<3	145 × 40 × 2 mm^3^
[3]	Metal-belt antenna -monopole2	700 MHz 2.7 GHz	NR	<3	185 × 40 × 2 mm^3^
[4]	Conformal antenna	690 to 960 MHz	2.03 dBi	<3	150 × 40 × 2 mm^3^
		1710 to 2170 MHz	3.33 dBi	<2	
		2400 to 2800 MHz	3.59 dBi	<2	
[5]	Metal-bezel antenna	1575.42 ± 1.023 MHz	9 dBi	<2	45 × 37 × 8 mm^3^
		2400–2484 MHz	12 dBi	<3	
[6]	Slot antenna	2.4 GHz	3.7 dBi	<2.4	23 × 23 × 10 mm^3^
[7]	Loop antenna	2400–2480 MHz	7 dBi	<2	44.8 × 44.8 × 6 mm^3^
		3400–3600 MHz	8 dBi	<2	
[8]	CMA-based antenna	1.575 GHz	NR	<2.4	58 × 58 × 8 mm^3^
		2.4 GHz	NR	<1.7	
		3.5 GHz	NR	<2.7	
[9]	Full-screen antenna	1.575 GHz	2 dBi	<2.4	34 × 28 × 10 mm^3^
		2.4 GHz	3 dBi	<2.5	
		3.5 GHz	4 dBi	<3	
		4.9 GHz	4 dBi	<1.5	
[10]	mm-wave antenna	25.5 GHz 38 GHz	9 dBi 8 dBi	<2 <2	6 × 6 × 1 mm^3^
[11]	mm-wave wearable antenna	28 GHz	1.45 dBi	<3.5	4.6 × 0.65 × 0.6 mm^3^
		38 GHz	4.85 dBi	<1.5	
[12]	Electrically small antenna	2.4 GHz	0.5 dBi	<1.2	17.4 × 17.4 × 1.6 mm^3^
[13]	Strap antenna	5.8 GHz	8 dBi	<2	45 × 15 × 1.6 mm^3^
[14]	Compact smartwatch antenna	2.4 GHz	5.5 dB	<1.5	38 × 38 × 5 mm^3^
		3.5 GHz	7.9 dBi	<1.5	
[15]	Flexible strap antenna	2.4 GHz	7.2 dBi	<2	220 × 44 × 6 mm^3^
[16]	PIFA	2.4 GHz	4 dBi	<2	45.3 × 18.97 × 1.84 mm^3^
This work	Wire inverted-F antenna	2.4 GHz	1.1 dBi	<1.5	37.4 × 31.2 × 5 mm^3^

NR—not reported or not directly comparable; EBG—electromagnetic band gap; CP—circular polarization; PIFA—planar inverted-F antenna.

## Data Availability

The original contributions presented in this study are included in the article. Further inquiries can be directed to the corresponding author.

## Abbreviations

The following abbreviations are used in this manuscript:

FDTD	Finite-Difference Time-Domain method
WBAN	Wireless Body Area Networks
GPS	Global Positioning System
5G	Fifth Generation of Mobile Communication Technology
SAR	Specific Absorption Rate
PIFA	Planar Inverted F Antenna
ISM	Industrial, Scientific, and Medical Band
IFA	Inverted F Antenna
VSWR	Voltage Standing Wave Ratio
LCD	Liquid Cristal Display
ABS	Acrylonitrile Butadiene Styrene
PCB	Printed Circuit Board
SMA	Sub Miniature Version A Connector
RSSI	Received Signal Strength Indicator

## References

[1] AnnA GlobalRnD. https://globalrnd.pl/anna/.

[2] Ashyap A.Y.I., Abidin Z.Z., Dahlan S.H., Majid H.A., Seman F.C. A Compact Wearable Antenna Using EBG for Smart-Watch Applications. Proceedings of the 2018 Asia-Pacific Microwave Conference (APMC).

[3] Zhao K., Ying Z., He S. Antenna Designs of Smart Watch for Cellular Communications by Using Metal Belt. Proceedings of the 9th European Conference on Antennas and Propagation (EuCAP 2015).

[4] Sayah S., Sarkis R. Design and Analysis of Conformal Antennas for Smart Watch. Proceedings of the 2017 Progress in Electromagnetics Research Symposium—Fall (PIERS-Fall).

[5] Zuo J., Wang Y., Xu F. (2025). Design of Dual-Band Antenna for Metal-Bezel Smartwatches with Negligible Wrist Effects Utilizing Coupled-Feeding Technique. IEEE Trans. Antennas Propag..

[6] Wu D., Cheung S.W., Li Q.L., Yuk T.I. Slot Antenna for All-Metal Smartwatch Applications. Proceedings of the 10th European Conference on Antennas and Propagation (EuCAP 2016).

[7] Li J., Wang Z., Wang J., Leach M., Pei R., Lim E.G., Luo Y. Integrated Loop Antenna for Smartwatch. Proceedings of the 2021 IEEE International Symposium on Antennas and Propagation and USNC-URSI Radio Science Meeting (APS/URSI).

[8] Zhang H.H., Gong L.F., Liu X.Z., Xu Y.X., Cheng G.S., Liu Y., Shi G.M., Zheng C., Han Y.J. (2023). Design of Low-SAR and High On-Body Efficiency Tri-Band Smartwatch Antenna Utilizing the Theory of Characteristic Modes of Composite PEC-Lossy Dielectric Structures. IEEE Trans. Antennas Propag..

[9] Xiao B., Wong H., Wu D., Yeung K.L. (2021). Design of Small Multiband Full-Screen Smartwatch Antenna for IoT Applications. IEEE Internet Things J..

[10] Bhadrvathi Ghouse P.S., Mane P.R., Thankappan Sumangala S., Kumar Puttur V., Pathan S., Jhunjhunwala V.K., Ali T. (2023). A Compact Dual-Band Millimeter Wave Antenna for Smartwatch and IoT Applications with Link Budget Estimation. Sensors.

[11] Ahmed M.I., Ahmed M.F. Design of 5G Smart Watch with Millimeter Wave Wearable Antenna. Proceedings of the 2019 7th International Japan-Africa Conference on Electronics, Communications, and Computations (JAC-ECC).

[12] Mhatre P., Joshi M. Electrically Small Wearable Tunable Antenna That Fits into Smartwatch Dial. Proceedings of the 2023 IEEE 8th International Conference for Convergence in Technology (I2CT).

[13] Rabhi R., Gahgouh S., Gharsallah A. (2022). Watchstrap Integrated Wideband Circularly Polarized Antenna Design for Smartwatch Applications. IET Microw. Antennas Propag..

[14] Duan Z., Xu L.-J. The Design of Smartwatch Antenna for Bluetooth and 5G Applications. Proceedings of the 2018 Asia-Pacific Microwave Conference (APMC).

[15] Kakaraparty K., Mahbub I. Smart-Watch Integrated Flexible Strap Antenna for Enhanced WiFi and Bluetooth Connectivity Applications. Proceedings of the 2024 IEEE International Symposium on Antennas and Propagation and INC/USNC-URSI Radio Science Meeting (AP-S/INC-USNC-URSI).

[16] Abdelhakim A., Cabedo-Fabrés M., Bataller M.F. PIFA Antenna for Smart Watch Application in the 2.4 GHz Band. Proceedings of the 2021 IEEE International Symposium on Antennas and Propagation and USNC-URSI Radio Science Meeting (APS/URSI).

[17] Chen W.-S., Yang C.-K., Sin W.-S. (2015). MIMO Antenna with Wi-Fi and Blue-Tooth for Smart Watch Applications. Proceedings of the 2015 IEEE MTT-S 2015 International Microwave Workshop Series on RF and Wireless Technologies for Biomedical and Healthcare Applications (IMWS-BIO).

[18] Hong S., Kwon J.-H. (2016). Numerical Calculation of Specific Absorption Rate for Smart-Watch with Planar Inverted F Antenna. Proceedings of the 2016 International Conference on Electromagnetics in Advanced Applications (ICEAA).

[19] Baloch B.A., Zahid Z., Khan A.A. (2022). Self-Decoupled Dual Band PIFA for Wi-Fi 6E Smartwatch MIMO Applications. Proceedings of the 2022 19th International Bhurban Conference on Applied Sciences and Technology (IBCAST).

[20] Luebbers R. (2006). XFDTD and Beyond-from Classroom to Corporation. Proceedings of the 2006 IEEE Antennas and Propagation Society International Symposium.

[21] Homsup N., Breakall J. (2010). Application of XFDTD and FEKO Program to the Analysis of Planar Antennas. Proceedings of the 2010 10th International Symposium on Communications and Information Technologies.

[22] Gosselin M.-C., Neufeld E., Moser H., Huber E., Farcito S., Gerber L., Jedensjö M., Hilber I., Gennaro F.D., Lloyd B. (2014). Development of a New Generation of High-Resolution Anatomical Models for Medical Device Evaluation: The Virtual Population 3.0. Phys. Med. Biol..

[23] Januszkiewicz Ł., Nowak I. (2024). Knitted Microwave Transmission Line for Wearable Electronics. Appl. Sci..

[24] (2017). Product Standard to Demonstrate the Compliance of Wireless Communication Devices with the Basic Restrictions and Exposure Limit Values Related to Human Exposure to Electromagnetic Fields in the Frequency Range from 30 MHz to 6 GHz: Hand-Held and Body-Mounted Devices in Close Proximity to the Human Body.

[25] Zhekov S.S., Franek O., Pedersen G.F. (2020). Dielectric Properties of Common Building Materials for Ultrawideband Propagation Studies [Measurements Corner]. IEEE Antennas Propag. Mag..

[26] Abd Rahman N.H., Yamada Y., Amin Nordin M.S. (2019). Analysis on the Effects of the Human Body on the Performance of Electro-Textile Antennas for Wearable Monitoring and Tracking Application. Materials.

[27] di Serio A., Buckley J., Barton J., Newberry R., Rodencal M., Dunlop G., O’Flynn B. (2017). Potential of Sub-GHz Wireless for Future IoT Wearables and Design of Compact 915 MHz Antenna. Sensors.

[28] Lin C.-H., Ito K. (2014). A Compact Dual-Mode Wearable Antenna for Body-Centric Wireless Communications. Electronics.

[29] Noghanian S. (2022). Dual-Band Wearable MIMO Antenna for WiFi Sensing Applications. Sensors.

[30] Shahzad M.A., Paracha K.N., Naseer S., Ahmad S., Malik M., Farhan M., Ghaffar A., Hussien M., Sharif A.B. (2021). An Artificial Magnetic Conductor-Backed Compact Wearable Antenna for Smart Watch IoT Applications. Electronics.

